# Development and Initial Assessment of a Novel Customized Deep Laceration Simulator for Suturing Training

**DOI:** 10.7759/cureus.32213

**Published:** 2022-12-05

**Authors:** Mithusa Sivanathan, Julia Micallef, Krystina M Clarke, Merieme Habti, Florence Bénard, Léamarie Meloche-Dumas, Erica Patocskai, Adam Dubrowski

**Affiliations:** 1 Health Sciences, Ontario Tech University, Oshawa, CAN; 2 Medical Pedagogy, University of Montreal, Montreal, CAN; 3 General Surgery, University of Montreal, Montreal, CAN; 4 Surgical Oncology, University of Montreal, Montreal, CAN

**Keywords:** three-dimensional printing, simulation-based learning, suturing, simulation-based medical education, deep cut, deep laceration, deep wound, simulator, training, 3d-printing

## Abstract

Suturing of different layers, such as deep lacerations, is a challenging clinical skill for residents. Currently, there is a lack of general suturing instructions and practice in undergraduate medicine curricula which would add to the education required during residency and could be impactful to patient safety. Therefore, in order to adequately prepare trainees for clinical practice, training in suturing needs to be made more robust and executable. One way to facilitate this is to provide easy access to equipment that can offer good educational value while allowing for adequate repetition of suturing deep lacerations outside of clinical settings, similar to how it has been done for superficial lacerations. Simulation-based medical education addresses this by training residents in healthcare skills in a safe and controlled environment. Our technical report aims to describe the development and initial evaluation of a deep laceration simulator designed to train residents in suturing. The simulator was made using additive manufacturing techniques such as three-dimensional printing and silicone. Feedback on the simulator was provided by Centre Hospitalier de l'Université de Montréal clinicians from various specialties and residents. The simulator was assessed mainly as being easy to use, durable, and having anatomically accurate characteristics. The main improvements suggested were to make the skin thinner, divide the epidermis and dermis, add a fascia, and create a looser and friable layer of fat. Overall, the respondents rated the simulator as a good educational tool with a few minor adjustments.

## Introduction

Wounds or cuts in the skin are normally the result of penetrating trauma or surgical incisions [1]. Deep wounds or lacerations, in particular, go beneath the skin through the fat or muscle layer and usually require medical attention [2]. Such injuries are routinely managed by medical professionals in the emergency department [3] and involve some kind of skin closure to promote healing [4]. Sutures are the primary method to approximate deep lacerations [5,6]. The choice of suture and technique depends on the type of wound, depth, degree of tension, and desired cosmetic result [7]. Due to the many considerations required to effectively treat deep lacerations, suturing of different layers is a challenging clinical skill for residents to gain proficiency in [8]. Currently, there is a lack of general suturing instructions and practice in undergraduate medicine curricula which would add to the education required during residency and could be impactful to patient safety [9,10]. On top of this, while minor lacerations are more commonly experienced in clinical settings, deep lacerations are often less common and thus less experienced by junior staff (C. Patey, personal communication, October 2, 2022). Therefore, in order to adequately prepare trainees to manage these deep lacerations, training in suturing needs to be made more robust and executable. One way to facilitate this would be to provide easy access to equipment that can offer good educational value while allowing for adequate repetition of suturing deep lacerations outside of clinical settings, similar to how it is done for superficial lacerations [11,12]. 

Simulation-based medical education addresses these gaps by allowing trainees to develop sound technical skills, such as deep laceration suturing, on simulators prior to performing this procedure on patients in clinical settings [13]. Simulators can be made using additive manufacturing techniques such as three-dimensional (3D) printing and silicone molding, resulting in inexpensive, anatomically accurate means to training [14]. The aim of this technical report is to describe the development and assessment of a deep laceration simulator. 

## Technical report

Context

Two collaborative teams with complementary areas of expertise co-designed a deep laceration simulator. A group of three designers, who are also graduate students, from maxSIMhealth laboratory, Ontario Tech University (OTU) located in Ontario, Canada comprised the development team, and a group consisting of one surgeon, four surgical trainees, one engineer, and one medical physicist from the Centre Hospitalier de l'Université de Montréal (CHUM) located in Montreal, Canada made up the clinical team. The deep laceration simulator was designed to train medical students who have little to no experience in suturing deep lacerations. 

Inputs and design process

Architecture of the Deep Laceration Simulator Mold

**Figure 1:**
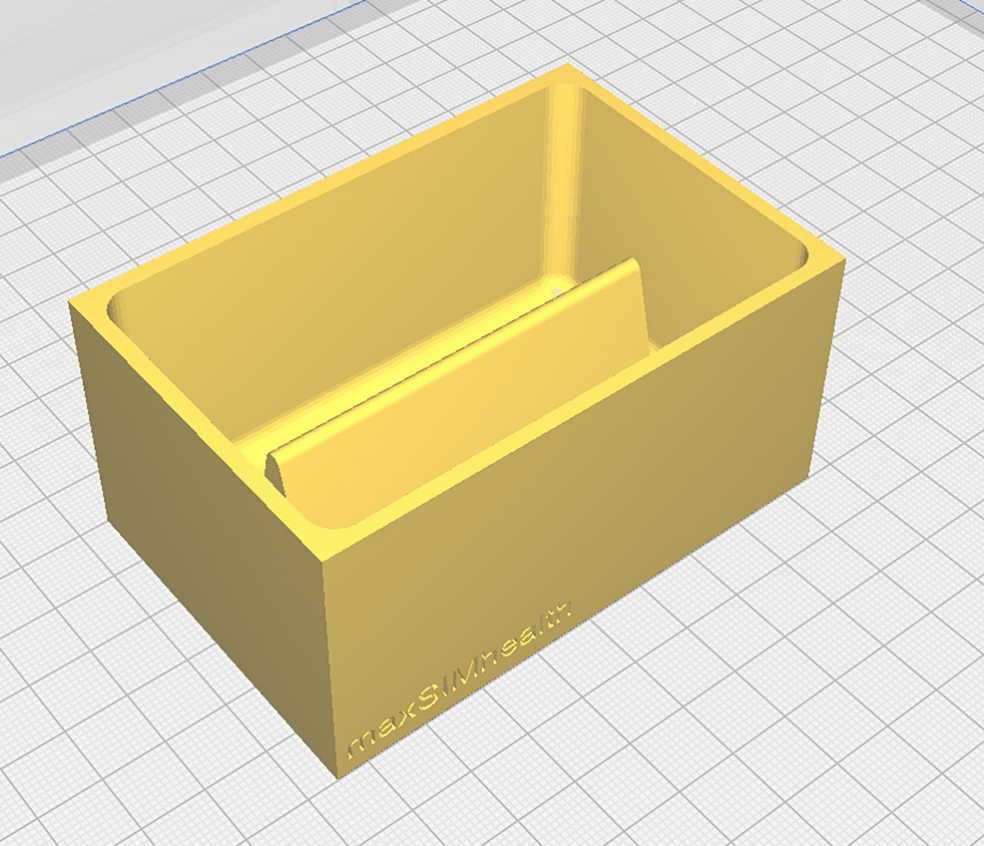
The digital 3D-rendering of the deep laceration simulator mold 3D: Three-dimensional

**Figure 2:**
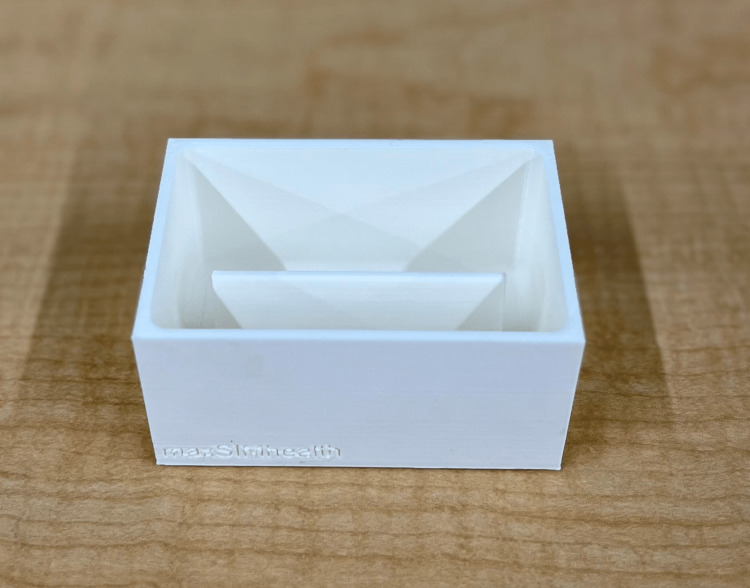
The 3D-printed deep laceration simulator mold 3D: Three-dimensional

The deep laceration simulator was designed ab initio by the development team. Using Fusion360™ (Autodesk Inc., San Rafael, CA), a digital 3D-rendering of the mold was made in the format of a stereolithography (.stl) file (Figure 1). It was then transferred to an Ultimaker S5 3D printer (Ultimaker B.V., Utrecht, Netherlands) using a secure digital card. Finally, the mold was printed using a white 3D-Fuel™ Pro polylactic acid (PLA) filament material (Fargo 3D Printing, Fargo, ND) (Figure 2). 

Using the mold, the deep laceration simulator was made using Dragon Skin™ NV 10 silicone (Smooth-On, Macungie, PA), Ecoflex™ 00-20 FAST silicone (Smooth-On, Macungie, PA), Ease Release™ 200 spray (Sculpture Supply Canada, Toronto, Ontario), Slacker™ silicone softener (Smooth-On, Macungie, PA), Silc-Pig™ coloring (Smooth-On, Macungie, PA), and power mesh (www.fabricland.com).

The digital file that was developed for this project is shared publicly here: https://github.com/maxSIMhealth/Deep_Laceration_Simulator. The use of all digital assets is bound by the terms and conditions of a Creative Commons Attribution-NonCommercial-ShareAlike 4.0 International Public License (CC BY-NC-SA 4.0). Subject to the terms and conditions of this Public License, the authors and creators hereby grant a worldwide, royalty-free, non-sublicensable, non-exclusive, irrevocable license to reproduce and share the licensed material, in whole or in part, for strictly non-commercial purposes; and produce, reproduce, and share adapted materials for non-commercial purposes only, under the same license. Please use this technical report as an acknowledgment for any research and description of educational activities that utilize any of these materials.

The materials included in this technical report are the intellectual property of many individuals (e.g., students, clinicians, engineers, researchers). The creator (A Dubrowski) and his institution (Ontario Tech University) do not warrant that these materials are complete, true, accurate, or non-misleading. By using these materials, the user agrees that the exclusions and limitations of liability set out in this disclaimer are reasonable. If the user does not think they are reasonable, they must not use these materials.

Selection of Materials of the Deep Laceration Simulator

The deep laceration simulator consists of three layers: skin, fat, and muscle. Each layer is made using silicone. Specifically, the skin layer was made using Ecoflex™ 00-20 FAST silicone as it is soft and flexible when it cures, similar to real skin. The fat and muscle layers were made using Dragon Skin™ NV 10 silicone which cures as harder and is more durable than skin, which is akin to real fat and muscle. The softness of the fat and muscle was adjusted using specific quantities of Slacker™. In between the layers of the deep laceration simulator, a piece of power mesh was embedded to prevent ripping of the silicone from repeated suturing as suggested in a previous development of an intestine model [15]. Finally, colouring using Silc-Pig™ was added to each layer of the deep laceration simulator to add realism: skin was made flesh tone (which can be adjusted to account for different skin colours); fat was made yellow; and muscle was made red. 

Process

Construction of the Deep Laceration Simulator

A specific sequence of steps were followed to construct the deep laceration simulator using its 3D-printed mold. The layers were assembled in the following order: skin, fat, and then muscle. 

First, the mold was sprayed with Ease Release™ 200 release agent spray and left to dry for approximately five minutes. During this waiting period, two pieces of power mesh were cut large enough to cover the opening of the mold. Next, the skin layer was prepared. Forty-five grams (g) of Ecoflex™ 00-20 FAST silicone were poured into a container and mixed until well-combined using a popsicle stick for about three minutes. Using a new popsicle stick, a pea-sized amount of flesh tone-coloured Silc-Pig™ silicone colouring was mixed into the silicone in the container until a uniform colour was achieved after one minute. Afterwards, the silicone mixture was poured into the mold to make the skin layer. The skin layer was left to cure for about 45 minutes in the mold, and then the first piece of mesh was placed on top.

Second, the fat layer was prepared. Thirty grams of Dragon Skin™ NV 10 silicone and 50g of Slacker™ silicone softener were poured into a container and mixed until well-combined using a popsicle stick for about three minutes. This is a 1:1.67 silicone-to-slacker ratio. Using a new popsicle stick, a pea-sized amount of yellow-coloured Silc-Pig™ was mixed into the silicone in the container until a uniform colour was achieved after one minute. Afterwards, the silicone mixture was poured into the mold, on top of the mesh and skin layer, to make the fat layer. The fat layer was left to cure for about 45 minutes in the mold, and then the second piece of mesh was placed on top.

**Figure 3:**
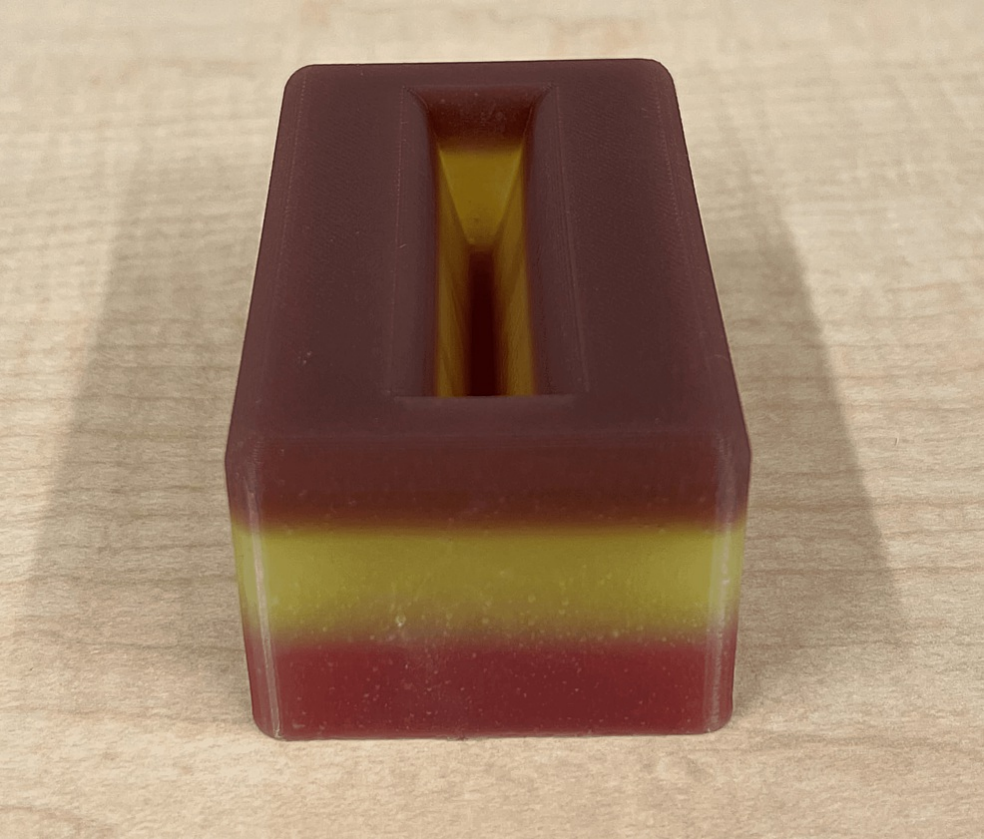
The deep laceration simulator

Finally, the muscle layer was prepared. Thirty grams of Dragon Skin™ NV 10 silicone and 30g of Slacker™ silicone softener were poured into a container and mixed until well-combined using a popsicle stick for about three minutes. This is a 1:1 silicone to slacker ratio. Using a new popsicle stick, a pea-sized amount of red-coloured Silc-Pig™ was mixed into the silicone in the container until a uniform colour was achieved after one minute. Afterwards, the silicone mixture was poured into the mold on top of the mesh and fat layer to make the muscle layer. The muscle layer was left to cure for about 45 minutes in the mold. Once cured, the entire deep laceration simulator was removed from the mold (Figure 3).

Assessment of the Deep Laceration Simulator

At CHUM, seven residents (training in a specialization in Canada) and four clinicians (have a certification of specialization in Canada, with +five years of experience in their specialization) from a variety of specialties, including general surgery, surgical oncology, and pathology, were asked to provide feedback on the design of the deep laceration simulator. There was no particular process in choosing who to request feedback from, simply any medical personnel at the institution were asked to offer insights if they had time and any experience in suturing. To accomplish this, three simulators were made at CHUM by the clinical team as instructed by the development team. These simulators along with their appropriate suturing equipment were provided to the residents and clinicians to practice or test out suturing (Video 1).

**Video 1:** Clinician suturing on the deep laceration simulator

All residents and clinicians (two with less than five years of experience and two with over 20 years of experience) then responded to an assessment survey adapted from the Michigan Standard Simulation Experience Scale template for the evaluation of the deep laceration simulator [16]. The survey aimed to gather (a) opinions regarding the simulator's representations of the anatomical features, (b) their perceptions of the simulator's potential to be used as an educational tool (i.e., perceived efficacy), and (c) their suggestions for any improvements that could be made to the simulator (Table 1). This content made up for 21 questions. Questions 4 to 6, 8 to 14, 16, and 19 prompted respondents to answer using a five-point scale, one denoting low levels of agreement and five indicating high levels of agreement. Question 17 asked respondents to rate the simulator as needing improvements on a four-point scale, one denoting extensive improvements required and four indicating no improvements necessary. All the other questions requested respondents to provide comments or to answer either yes or no. 

**Table 1:** Assessment survey questions for the deep laceration simulator

Question #	Deep Laceration Simulator Survey Questions				
DEMOGRAPHICS					
1	What is your specialty?				
2	If you are a student, what year of your program are you in?				
3	If you are a practicing physician, how long have you been practicing for?				
SELF-EFFICACY					
4	This model helped improve my KNOWLEDGE on the procedure in scope				
5	This model helped improve my CONFIDENCE in performing the procedure in scope				
6	This model helped improve my ABILITY in performing the procedure in scope				
7	Comment/suggestions regarding the model that may improve self-efficacy.				
FIDELITY					
8	This deep suture pad has anatomically accurate characteristics/features.				
9	On a scale of 1 to 5, how accurate did the skin layer feel?				
10	On a scale of 1 to 5, how accurate did the fat layer feel?				
11	On a scale of 1 to 5, how accurate did the muscle layer feel?				
12	Comments/suggestions to improve fidelity of the deep suture pad.				
13	Comments/suggestions to improve the functionality of the deep suture pad.				
14	On a scale of 1 to 5, how difficult was it to use the model?				
15	Comments/suggestions to make the simulator less difficult to use.				
OVERALL RATING					
16	Overall, the deep suture pad was a helpful training tool for the procedure in scope.				
17	For the evaluation of the model...				
18	Comments/suggestions to improve the deep suture pad overall.				
19	Other than the simulator used today, have you used a deep suture pad in the past?				
20	If another deep suture pad has been used in the past, what was the simulator called and/or can you provide a quick description of the simulator?				
21	If you have used another deep suture pad in the past, how does that one compare to the one used today?				

Averages and standard deviations (SDs) were calculated manually and used to interpret the responses from the questions that used a five-point scale. Questions that scored 3.5 and above in average indicated a positive response to the question while below 3.5 in average indicated a negative response to the question. Questions that had a SD of 1 or higher indicated low levels of agreement among the respondents while a SD below 1 indicated high levels of agreement. 

Products/outcomes

Costs

Table 2 shows the breakdown of all costs associated with the manufacturing of one deep laceration mold and simulator. The cost of the mold is a one-time expense as one can be used multiple times to create the simulators. The silicone used to make the simulator is a consumable cost. Currently, there is not enough evidence to estimate how many sutures a single simulator can withstand. All cost estimates are in Canadian dollars (CAD), including local taxes.

**Table 2:** Cost breakdown of the deep laceration mold and simulator *It includes two pieces of 54cm^2^ 9cm x 6cm pieces of power mesh that go on top of the muscle and fat layers. CAD: Canadian dollars Manufacturer details 3D-Fuel™ Pro PLA filament: Fargo 3D Printing, Fargo, ND Dragon Skin™ NV 10 silicone: Smooth-On, Macungie, PA Ecoflex™ 00-20 FAST silicone: Smooth-On, Macungie, PA Slacker™ silicone softener: Smooth-On, Macungie, PA Power mesh: www.fabricland.com

Material	Amount Used	Cost (in $ CAD with taxes)
3D-Fuel™ Pro PLA filament	51 grams	1.73
Dragon Skin™ NV 10 silicone	60 grams	3.92
Ecoflex™ 00-20 FAST silicone	45 grams	2.51
Slacker™ silicone softener	80 grams	5.24
Power mesh	96 cm^2^*	0.06
	TOTAL COST	13.46

Results/User Feedback Assessments

Eleven out of eleven residents and clinicians completed the survey after they used the deep laceration simulator, for a response rate of 100%. Their averages and results for questions using scales are presented in Table 3. Most participants felt that their knowledge and confidence in suturing a deep laceration improved. The simulator was assessed mainly as being easy to use, durable, and having anatomically accurate characteristics. Some suggestions were provided through open-ended questions on how to improve the deep laceration simulator (Table 4). Most of the improvements focused on the feeling or the texture of the model itself in comparison to real life deep laceration and this was also reflected in the statistics provided by Questions 9 and 10. Overall, the majority of respondents rated the deep laceration simulator as a helpful training tool to learn suturing.

**Table 3:** Average and SD of responses to questions using scales in the assessment survey *It requires extensive improvements before it can be considered for use in training. **It requires minor adjustments before it can be considered for use in training. ***It can be used in training but should be improved slightly. ****It can be used in training with no improvements made. SD: Standard deviation

Deep Laceration Pad Quantitative Survey Data								
Question #	Scale 1 (strongly disagree) to 5 (strongly agree)					Total Responses	Average Response	Standard Deviation
	1	2	3	4	5			
4	0	0	2	4	3	9	4.11	0.78
5	0	0	0	7	2	9	4.22	0.44
6	0	0	1	4	4	9	4.33	0.71
8	0	1	3	4	3	11	3.82	0.98
	Scale 1 (not accurate) to 5 (very accurate)							
	1	2	3	4	5			
9	0	2	2	5	2	11	3.64	1.03
	Scale 1 (not well) to 5 (very well)							
	1	2	3	4	5			
10	1	2	2	5	1	11	3.27	1.19
	Scale 1 (not durable) to 5 (very durable)							
	1	2	3	4	5			
11	0	1	1	5	4	11	4.09	0.94
	Scale 1 (very difficult) to 5 (not difficult)							
	1	2	3	4	5			
14	0	0	0	2	9	11	4.82	0.4
	Scale 1 (strongly disagree) to 5 (strongly agree)							
	1	2	3	4	5			
16	0	0	0	5	6	11	4.55	0.52
	Option 1*	Option 2**	Option 3***	Option 4****				
17	0	2	5	4		11	3.18	0.75

**Table 4:** Comments provided through the open questions in the assessment survey

Deep Laceration Simulator Qualitative Survey Data	
Question #	Comments
7	I think for the medical students and also low-grade residents in surgery specialties, it is very good; good model that is reusable and stays on the table while suturing; very good model
12	The fat layer should be looser to be identical to the real fat tissue in the patient; divide epidermis and dermis; thinner skin layer; fat easy to suture which is good for teaching but not realistic; it is useful to practice the technique but not very realistic anatomically; would recommend making the skin layers thinner; the texture of the different layers can be made more realistic; different size; slightly more forgiving than real times
13	Good; skin too large in height, fat not friable enough, needs a dermis and a fascia; add a muscle fascia and a harder superficial dermis
15	Maybe if the size of the simulator would be longer it would be better; I would suggest to make the layers more grabbable, maybe by separating them
18	The texture of yellow part is NOT as loose as real simulation; make the proportions more realistic; none
20	The deeper layer should be more flexible and fragile as well to mimic better the fat tissue; too light, does not stick on the table, breaks easily
21	This one is much better than the previous one; very similar, the other has a thinner layer but this one = more durable; it was less durable than deep suture pad tried today; the pad stays in place; the pad is reusable, packable, easy clean, and aesthetically pleasing

The responses from the open-ended questions of the assessment survey are presented in Table 3. The deep laceration simulator was generally perceived to be a good model to improve self-efficacy for residents and medical students. With regard to improving fidelity of the model, there were remarks made on the size and texture of the specific anatomical components. Requests were made to make the skin thinner and divide the epidermis and dermis, and it was noted that the fascia was missing in the model. Finally, a comment was also made to make the fat slightly looser and friable to better simulate real fat. Lastly, the respondents felt that the deep laceration simulator was superior overall in comparison to other models they have tried in the past because it was heavier, gripped onto the table, and did not break easily. In addition, respondents highlighted that the deep laceration model was packable, easy to clean, and aesthetically pleasing. In summary, the open-ended questions also revealed that the model is a good training tool. 

## Discussion

The aim of this technical report was to describe the development and initial evaluation of a deep laceration simulator designed to provide residents an opportunity to practice suturing a deep laceration. A group of 11 residents and clinicians rated the deep laceration simulator high in educational quality. The prevailing opinion was that the model was easy to work with, long-lasting, and able to teach suturing deep lacerations. Minor adjustments could be made to the model to increase its fidelity. Respondents mainly commented on the proportions and consistency of certain anatomical features. 

There were a few shortcomings pertaining to the evaluation process employed for the deep laceration simulator. Only 11 individuals provided feedback on the simulator, of which only four were clinicians and seven were residents. Residents have less experience than clinicians in most clinical skills since they are still in the learning phase, so they would not be able to gauge the educational value of the simulator as well as experienced clinicians would. However, residents prior to suturing on patients will have had experience working with other simulators in the past, but more recently in comparison to practicing clinicians (i.e., those practicing for 20 years) at the time of the evaluation. Therefore, one could argue that residents would be better able to make a comparison between the deep laceration simulator presented in this technical report to other similar simulators. Considering these two above-mentioned points, future improvements to the evaluation process for simulators would be to have a balance of the type and number of resident and clinician respondents.

In-person, verbal feedback by an experienced clinician specializing in surgical oncology and a general surgery resident revealed that the deep laceration simulator was too thick. With regard to the shape of the deep laceration, a suggestion was made to change it from a rectangle to an elliptical as cuts never occur in that form in real life. Anatomically speaking, the fascia, a thin layer between the muscle and the fat, was missing. The fat was noted to be too dense and should be made more jelly-like. Additionally, lobules could be made and dispersed throughout the fat layer to add realism. Finally, the muscle was deemed acceptable. Overall, the clinician and resident who provided verbal feedback rated the deep laceration simulator as a good teaching tool. When comparing the verbal feedback process to the surveys that collected feedback, generally the verbal feedback was more detailed than the survey feedback. In fact, most of the open-ended questions on the survey yielded a few words as a response without much elaboration. This is because those that completed the surveys did so between operations or at the end of the workday meaning they were restrained on time to provide in-depth responses to the survey questions. Based on this experience, it might be more appropriate to conduct focus group interviews to gather detailed feedback on possible improvements to the deep laceration simulator.

## Conclusions

This technical report demonstrates that a deep laceration simulator can be created using additive manufacturing techniques and that it is a helpful tool for learning the suturing technique. This simulator can aid to address the gap in the medical education landscape where there are not enough opportunities for practice provided during undergraduate curricula on suturing, let alone deep lacerations, which may be an inconvenience during residency and eventual clinical practice. With a few small adjustments, this approach could offer surgical trainees a reliable and realistic way to practice their suturing skills on deep lacerations.

## References

[1] (2022). Lacerations. https://www.hopkinsmedicine.org/health/conditions-and-diseases/lacerations.

[2] (2022). Wound Home Skills Kit: Lacerations & Abrasions. https://www.facs.org/media/x04ddnc5/wound_lacerations.pdf.

[3] Mankowitz SL (2017). Laceration management. J Emerg Med.

[4] Rose J, Tuma F (2021). Sutures and Needles. StatPearls [Internet].

[5] Forsch RT (2008). Essentials of skin laceration repair. Am Fam Physician.

[6] Byrne M, Aly A (2019). The surgical suture. Aesthet Surg J.

[7] Azmat CE, Council M (2022). Wound Closure Techniques. StatPearls [Internet].

[8] Emmanuel T, Nicolaides M, Theodoulou I, Yoong W, Lymperopoulos N, Sideris M (2021). Suturing skills for medical students: a systematic review. In Vivo.

[9] Gallagher PO, Bishop N, Dubrowski A (2020). Investigating the perceived efficacy of a silicone suturing task trainer using input from novice medical trainees. Cureus.

[10] Manning EP, Mishall PL, Weidmann MD (2018). Early and prolonged opportunities to practice suturing increases medical student comfort with suturing during clerkships: suturing during cadaver dissection. Anat Sci Educ.

[11] Gershuni V, Woodhouse J, Brunt LM (2013). Retention of suturing and knot-tying skills in senior medical students after proficiency-based training: results of a prospective, randomized trial. Surgery.

[12] Williams TP, Snyder CL, Hancock KJ (2020). Development of a low-cost, high-fidelity skin model for suturing. J Surg Res.

[13] Hammoud MM, Nuthalapaty FS, Goepfert AR (2008). To the point: medical education review of the role of simulators in surgical training. Am J Obstet Gynecol.

[14] Bartellas M (2016). Three-dimensional printing and medical education: a narrative review of the literature. UOJM.

[15] Habti M, Bénard F, Arutiunian A (2021). Development and learner-based assessment of a novel, customized, 3D printed small bowel simulator for hand-sewn anastomosis training. Cureus.

[16] Seagull FJ, Rooney DM (2014). Filling a void: developing a standard subjective assessment tool for surgical simulation through focused review of current practices. Surgery.

